# Consistency of Suspended Particulate Matter Concentration in Turbid Water Retrieved from Sentinel-2 MSI and Landsat-8 OLI Sensors

**DOI:** 10.3390/s21051662

**Published:** 2021-02-28

**Authors:** Hanghang Wang, Jie Wang, Yuhuan Cui, Shijiang Yan

**Affiliations:** 1 College of Resources and Environmental Engineering, Anhui University, Hefei 230601, China; x18301078@stu.ahu.edu.cn (H.W.); yanshij@ahu.edu.cn (S.Y.); 2 Anhui Province Key Laboratory of Wetland Ecosystem Protection and Restoration, Anhui University, Hefei 230601, China; 3 College of Science, Anhui Agricultural University, Hefei 230036, China; cuiyh@ahau.edu.cn

**Keywords:** MSI sensor, OLI sensor, remote sensing, suspended particulate matter, turbid water, consistency

## Abstract

Research on the consistency of suspended particulate matter (SPM) concentration retrieved from multisource satellite sensors can serve as long-time monitoring of water quality. To explore the influence of the atmospheric correction (AC) algorithm and the retrieval model on the consistency of the SPM concentration values, Landsat 8 Operational Land Imager (OLI) and Sentinel 2 MultiSpectral Imager (MSI) images acquired on the same day are used to compare the remote sensing reflectance (Rrs) SPM retrieval values in two high-turbidity lakes. An SPM retrieval model for Shengjin Lake is established based on field measurements and applied to OLI and MSI images: two SPM concentration products are highly consistent (*R*^2^ = 0.93, Root Mean Squared Error (RMSE) = 20.67 mg/L, Mean Absolute Percentage Error (MAPE) = 6.59%), and the desired results are also obtained in Chaohu Lake. Among the four AC algorithms (Management Unit of the North Seas Mathematical Models (MUMM), Atmospheric Correction for OLI’lite’(ACOLITE), Second Simulation of Satellite Signal in the Solar Spectrum (6S), Landsat 8 Surface Reflectance Code & Sen2cor (LaSRC & Sen2cor)), the two Rrs products, as well as the final SPM concentration products retrieved from OLI and MSI images, have the best consistency when using the MUMM algorithm in SeaWIFS Data Analyst System (SeaDAS) software. The consistency of SPM concentration values retrieved from OLI and MSI images using the same model or same form of models is significantly better than that retrieved by applying the optimal models with different forms.

## 1. Introduction

As the main component of case-II water, suspended particulate matter (SPM) concentration is an important parameter used in the assessment of aquatic ecosystems and environmental impact of water [1,2,3]. It also plays a key biogeochemical role in aquatic ecosystems [4]. SPM transportation and accumulation have significant effects on aquatic ecosystems and human activities [5]. Therefore, monitoring the temporal and spatial variations in the SPM concentration in lakes is important since it allows to understand the dynamics of suspended particulates, as well as the structure and function of aquatic ecosystems, and in the management and protection of aquatic ecosystems [1,2,3,4,5,6,7].

Monitoring of the SPM concentration in inland lakes and estuaries is usually performed via field surveys and laboratory measurements [8,9,10]. However, it is difficult to achieve high-density spatial monitoring of the SPM concentrations in large water bodies using these methods as they are time-consuming and expensive [11]. Satellite remote sensing technology can provide global coverage and allow the monitoring of specific areas. Remote sensing enables large-scale observations of the spectral reflectance of water in the visible and near-infrared region, which can be used to map the SPM concentration in turbid waters around the world [5,12]. Satellite remote sensing technology has, therefore, become an important tool for monitoring the water quality of large and medium-sized lakes.

Although ocean color satellite sensors (such as Sea-viewingWideFieldSensor (SeaWiFS), Moderate Resolution Imaging Spectroradiometer (MODIS), MEdium Resolution Imaging Spectrometer (MERIS), and Geostationary Ocean Color Imager (GOCI)) are equipped with special spectral bands for observing the bio-optical properties of water bodies, the low spatial resolution (250 m to 1 km) of these bands limits their capability to monitor inland water bodies at a small scale [13]. Land-monitoring satellites, such as the Landsat and Sentinel-2 satellites, can provide imagery with a high spatial resolution (10–30 m), which can be used to monitor the water quality parameters of inland lakes and rivers. Launched on 11 February 2013, Landsat-8 carries the Operational Land Imager (OLI), which has a spatial resolution of 30 m and a temporal resolution of 16 days. Launched on June 2015, Sentinel-2 carries the MultiSpectral Imager (MSI), which has a spatial resolution of 10–60 m and a temporal resolution of 5 days, making it the first satellite to systematically observe the world’s coastal zones at both high spatial and high temporal resolutions. Researchers have used Landsat and Sentinel-2 imagery to map the surface SPM concentration of large rivers and lakes around the world, including the Mississippi River [14], Loire River [5], Guadalquivir River [15], Dongting Lake [7], Poyang Lake [12], and Yangtze River [16]. However, due to the influence that frequent cloudy and rainy weather in middle and low latitudes has on the quality of satellite imagery, the use of a single type of optical data has certain limitations in terms of effectively capturing the highly dynamic variations in SPM concentration in inland waters [11]. A combination of Landsat-8 and Sentinel-2 images has a temporal resolution of ~3.8 days, which is a short revisit cycle [17]. Therefore, the combined use of Landsat and Sentinel-2 images to map the dynamic changes in SPM in inland water bodies has been a trend in recent studies.

In recent years, combination of Landsat-8 and Sentinel-2 data has been used in the monitoring of various water quality parameters of lakes and rivers. These parameters have included the dissolved organic carbon concentration [18], chlorophyll-a concentration [19,20], turbidity [20], water clarity [21], and SPM concentration [13]. Ensuring the consistency of water quality values retrieved from MSI and OLI images is a prerequisite for integrating these two remote sensing data sources, so as to carry out the long-term monitoring of aquatic systems. The retrieved water quality values are affected by atmospheric correction of remote sensing images, the retrieval models and other factors, and it is vital to ensure the consistency of remote sensing reflectance derived from OLI and MSI images. However, in these studies, only the consistency of the retrieved values of the water quality parameters for a single area was considered, and the different links in the retrieval process, which significantly limit the transferability of remote sensing products and the range of applications in which they can be used, were not analyzed.

The general objectives of this study were to (1) compare the SPM concentration values retrieved from Sentinel-2 MSI and Landsat-8 OLI images acquired on the same day, and (2) explore the influence of the atmospheric correction algorithm and the retrieval model on the consistency of the SPM concentration values. To do this, the combinations of Landsat-8 and Sentinel-2 images were used to analyze the consistency of SPM remote sensing products for two high-turbidity lakes in the middle and lower reaches of the Yangtze River of China. The influence of the atmospheric correction algorithm and retrieval models used (same model, same form of model, or optimal model with different forms) on the consistency of the SPM concentration values retrieved from MSI and OLI images was explored. The results would provide a basis for the seamless integration of Landsat-8 OLI and Sentinel-2 MSI imagery for performing long-term monitoring of water quality parameters in rivers and lakes.

## 2. Materials and Methods

### 2.1. Study Areas

**Figure 1:**
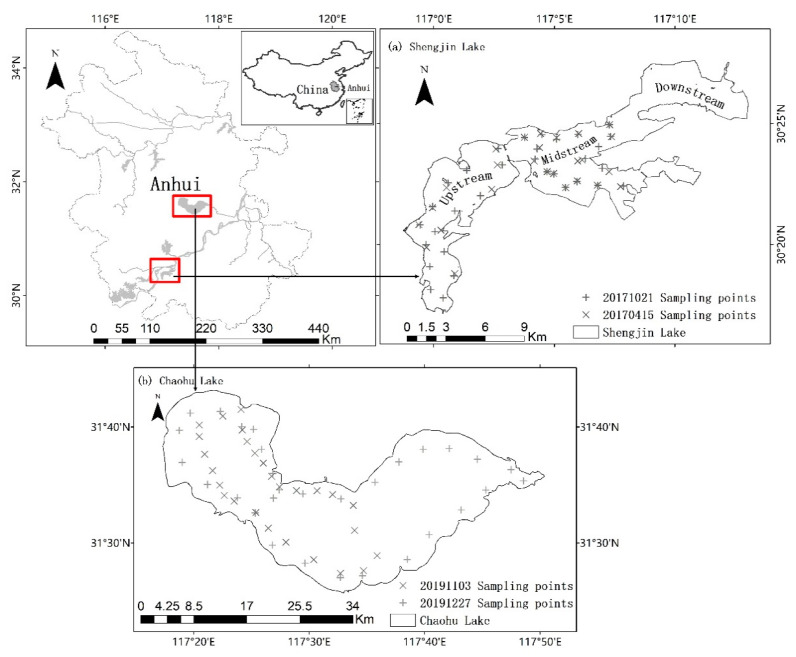
Location of study area and sampling sites. (**a**) Shengjin Lake, (**b**) Chaohu Lake.

Shengjin Lake and Chaohu Lake were selected as the study areas, and both are located in the middle-to-lower reaches of the Yangtze River in Anhui Province, China (Figure 1). Shengjin Lake (116°55′–177°15′ E, 30°15′–30°30′ N) is a shallow lake located on the south side of the Yangtze River in Chizhou City, Anhui Province. The lake comprises three parts: the upstream, the midstream, and the downstream, and it has a total area of about 133 km^2^ during the wet season. The average SPM concentration and average chlorophyll-a concentration in the lake are 62.28 mg/L and 6.83 μg/L respectively, meaning that the lake can be classified as a turbid lake dominated by SPM. Chaohu Lake (117°17′–117°51′ E, 31°25′–31°43′ N), located on the north bank of the Yangtze River, is one of the five largest freshwater lakes in China and has a total area of 775 km^2^. The average SPM concentration and average chlorophyll-a concentration in the lake are 32.53 mg/L and ~138.77 μg/L, respectively; thus, the lake can be classified as a turbid lake dominated by chlorophyll-a.

### 2.2. Field Measurements

Two field surveys were conducted at Shengjin Lake: one on April 14–15 and another on October 21–22, 2017, and a total of 62 samples were collected from the lake surface (Figure 1a). Two field surveys were also conducted at Chaohu Lake: one on November 3 and another on December 27, 2019, and a total of 58 samples were collected (Figure 1b). The remote sensing reflectance (Rrs) of the water surface was detected using an AvaField-1 portable surface spectrometer with a spectral range of 350–1050 nm (Avantes Company, Netherlands). Using the above-water surface measurement method [22], the measurements were recorded at an observation azimuth angle of 135° and an observation zenith angle of 40°. The remote sensing parameters that were measured included the irradiance of a standard gray plate, sky radiance, and water-leaving radiance. Simultaneously, the water clarity and turbidity were determined using a standard 20 cm diameter Secchi disk made in China and an HACH 2100Q turbidity meter made in USA, respectively, while the coordinates of each sample were recorded by a hand-held Global Positioning System (GPS) receiver (Trimble GEOXH2008). Water samples (500 mL) were collected at a depth <30 cm using a suction pump. These samples were taken back to the laboratory for determination of the SPM concentration, which were pre-weighed by Whatman Glass fiber filtersFiltration (GF/F) glass microfiber filters (diameter 47 mm, pore size 0.45 μm), then the filters were dried at 40 °C for 48 h and reweighed [23]. The preprocessing of the spectral data and calculation of the water surface Rrs were performed following the method described by Wang [24]. The measured Rrs (λ) was calculated using Equation (1):

(1) $$\;\mathrm{Rrs}\left(\lambda \right)=\frac{L_{w}-{\rho L}_{\mathrm{sky}}}{{\pi L}_{p}/\rho_{p}}$$

where L_w_ is the radiance measured above the water surface, L_sky_ is the radiance of the sky measured by pointing the spectrometer sensor at the sky at a zenith angle of 45°, ρ_p_ is the reflectance of the standard gray plate, L_p_ is the downward solar radiation received by the spectrometer above the gray plate, and ρ is the dimensionless air–water reflection (a constant value of 0.025 was used in this study [24]).

### 2.3. Satellite Images Acquisition and Preprocessing

A Sentinel-2 MSI image of Shengjin Lake acquired on 24 October 2017, as well as MSI and OLI images acquired on 23 November 2019, all of which were of good quality and with no cloud cover, were used in this study. For Chaohu Lake, a Sentinel-2 MSI image of Shengjin Lake acquired on 27 December 2019, as well as MSI and OLI images acquired on 23 November 2019, were also of good quality with thin cloud cover. Landsat-8 OLI images were downloaded from the remote sensing image database of the US Geological Survey (USGS) (http://earthexplorer.usgs.gov/ (accessed on 25 May 2020)), and the Sentinel-2 MSI images were downloaded from the European Space Agency site (https://sentinel.esa.int (accessed on 28 May 2020)).

SeaWIFS Data Analyst System (SeaDAS) 7.5 software was used to preprocess remote sensing images. The preprocessing included geometric and atmospheric correction. The Management Unit of the North Seas Mathematical Models (MUMM) algorithm was one of the atmospheric correction methods used [25]. The MUMM algorithm is an atmospheric correction method that can be applied to case-II water and is an extension of Gordon’s standard atmospheric correction algorithm [26]. The MUMM algorithm assumes that the ratio of the aerosol scattering rate and the water-leaving emissivity at 765 and 865 nm in the study area have fixed values, and it also assumes that the ratio of the water reflectance at 765 nm to that at 865 nm, as corrected by the atmospheric diffuse transmittance, has a fixed value. The radiative transfer equation is solved, and the atmospheric correction is performed according to the standard algorithm [27,28].

The water bodies in the imagery were extracted using the modified automatic water extraction index (MAWEI) algorithm (Equation (2)) [29]. This is an improved version of the automatic water extraction index (AWEI) and further enhances the difference between water and other ground objects. Compared to the object-oriented methods, AWEI and the modified normalized difference water index, the MAWEI algorithm extracts water bodies more accurately. The coastline effect caused by mixed pixels at the edges of water bodies can affect dark targets, and this is especially important in the case of inland water bodies. To reduce this interference and minimize the subpixel variability, the MAWEI algorithm masks and discards two pixels near the coast [30]. The equation used to calculate the MAWEI is:

(2) MAWEI=5×(B3−B8a)+(B2+B4−4×B12)

where B2, B3, B4, B8a, and B12 are the Rrs at the blue, green, red, near-infrared, and shortwave infrared bands of the MSI sensor, respectively.

### 2.4. SPM Retrieval Model Development

Firstly, the Rrs at the spectral bands of MSI and OLI sensors in the range of 400–900 nm are simulated before constructing the retrieval models. To simulate the Rrs at the bands in two sensors, the water spectra data and the relative spectral responses of the MSI and OLI sensors were combined to simulate the Rrs in the different bands. The simulated Rrs was calculated according to Equation (3) [31]:

(3) $$\mathrm{Rrs}=\frac{\int_{\lambda_{2}}^{\lambda_{1}}\mathrm{fi}\left(\lambda \right)\mathrm{Rrs}\left(\lambda \right)d\lambda }{\int_{\lambda_{2}}^{\lambda_{1}}\mathrm{fi}\left(\lambda \right)d\lambda }$$

where Rrs is the simulated Rrs at the spectral bands, Rrs(λ) is the Rrs of the measured water spectra, fi(λ) is the relative spectral response of the sensor, and λ_1_ and λ_2_ are the wavelengths corresponding to the lower and upper ends of the sensor bands, respectively.

Then Pearson correlation analysis between different band combinations (the mathematical operations of addition, subtraction, multiplication, and division) and the measured SPM concentration was conducted. After sorting the measured SPM values, two-thirds of the samples were randomly selected as training samples. Taking the band reflectance combination with correlation coefficient R greater than 0.8 (Table 1) as the independent variables, and the measured suspended solids concentration as the dependent variable, different forms of inversion models are established according to the partial least square method. The model forms include polynomial model, exponential model, logarithmic model, power function model, etc. These retrieval models were used to predict the SPM concentration in the lakes.

The accuracy assessment of the retrieval models that were used in this study was performed based on the ground observations. The remaining one-third of the samples were used as test samples to validate the accuracy of the retrieval models using the ground observations. To reduce the effect of the geographic location and spatial resolution of the imagery, the retrieved SPM values were calculated using an averaging 3 × 3-pixel window centered at the positions of the samples. In addition, pixels whose values changed at a rate of greater than 20% within the 3 × 3 window were eliminated to reduce the effect of spatial heterogeneity of the SPM in the water [32]. Finally, the results of the evaluation of the calibration accuracy of the retrieval model, and that of ground verification accuracy, were integrated. From this, the best model was determined.

### 2.5. Evaluate the Consistency of SPM Remote Sensing Products

After SPM retrieval models are applied to Landsat-8 and Sentinel-2 images, the consistency of the retrieved values of the SPM concentration is evaluated. First, the SPM values retrieved from Sentinel-2 images were resampled to 30 m using the extended geospatial data abstraction library in Python, georeferenced with Landsat-8 images on the same day; then, the correlation between the SPM values retrieved from Landsat-8 and Sentinel-2 images, and the difference between them (OLI minus MSI), were analyzed, to verify the consistency between these two SPM concentration products. The indices used to evaluate the consistency of the remote sensing products in our paper include the coefficient of determination (R^2^), root mean squared error (RMSE), and mean absolute percentage error (MAPE).

## 3. Results

### 3.1. Consistency of Rrs Values Derived from OLI and MSI Images

The Rrs values for bands B1–B4 and B8a of the Sentinel-2 MSI image in Shengjin Lake acquired on 23 November 2019 were reconstructed and resampled to 30 m. The Rrs values for these five bands were read pixel-by-pixel and compared to those for the Landsat-8 OLI image from the same day to analyze their consistency.

**Figure 2:**
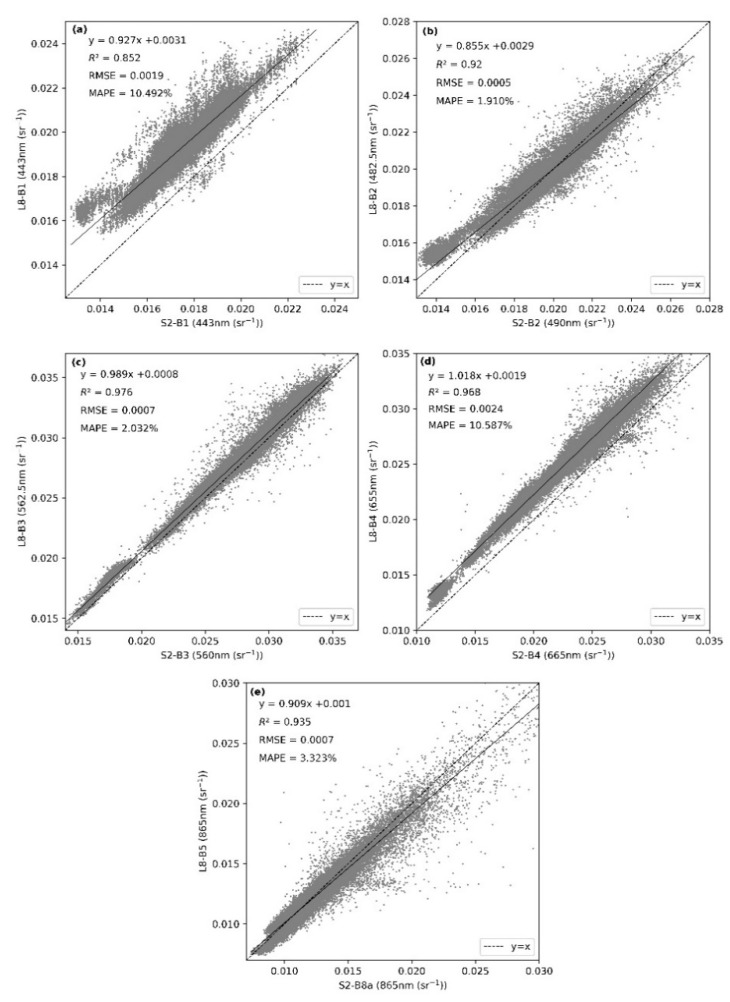
Consistency of the remote sensing reflectance (Rrs) values derived from MSI and OLI images in Shengjin Lake. (**a**) Coastal blue band, (**b**) Blue band, (**c**) Green band, (**d**) Red band, (**e**) Near-infrared band.

From the Rrs consistency results (Figure 2), it can be seen that the Rrs values for the green, blue, and near-infrared bands of the MSI and OLI images are distributed on both sides of the y = x line, which means that the consistency is good. For the blue band, RMSE = 0.0005 and MAPE = 1.910%, for the green band, RMSE = 0.0007 and MAPE = 2.032%, and for the NIR band, RMSE = 0.0007 and MAPE = 3.323%. However, from the line of best fit, Rrs for the NIR band of the MSI sensor is significantly higher (a difference of 0.015) than that of the OLI, mainly because these Rrs values are higher and closer to the values for the land surface. Zhang et al. reported that the Rrs values for MSI images of land are higher than those of OLI images and that these differences may be caused by environmental conditions or the spatial resolution, rather than being related to sensor features [20,33,34,35]. It can also be seen from the figure that the RMSE and MAPE values for the red and coastal blue bands are relatively large (for both bands, the RMSE is greater than 0.0015, and the MAPE value is greater than 10%). In addition, the OLI Rrs is significantly higher than the MSI Rrs. This difference between the coastal blue band Rrs for these two images is mainly related to the error produced by the MUMM atmospheric correction algorithm [28], whereas, for the red band, the difference is mainly a result of the difference in the spectral range, central wavelength, and spectral response function of these two sensors.

### 3.2. SPM Retrieval Model Developed for OLI and MSI Sensors

**Figure 3:**
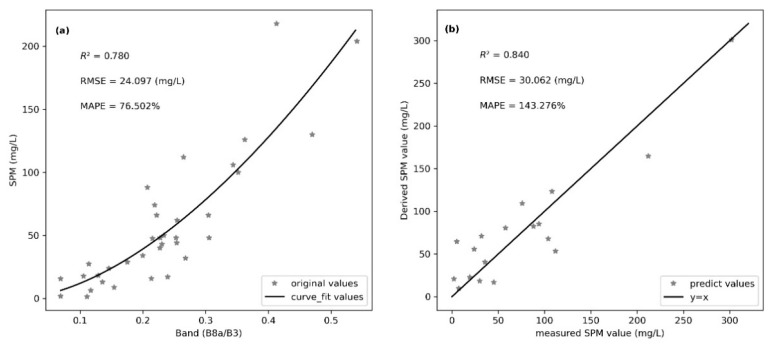
Accuracy assessment of SPM retrieval model for OLI and MSI sensors in Shengjin lake. (**a**) Calibration accuracy, (**b**) validation accuracy.

To ensure the transferability of the SPM retrieval model between the OLI and MSI sensors, an SPM retrieval model common for OLI and MSI sensors was developed in Shengjin Lake using the partial least squares method, based on the synchronous field measurement of Rrs and SPM values. Then, the accuracy of the retrieval model was comprehensively evaluated using the three indicators (R^2^, RMSE, and MAPE) of calibration accuracy and validation accuracy. The results showed that the model with the highest accuracy was the power function model given by y = 610.210 x^1.705^, where x is B8a/B3 (Figure 3).

Figure 3 shows that, for ground observations in Shengjin lake, the calibration accuracy and validation accuracy of the power function model above are higher than other types of models. The calibration accuracy gives an R^2^ of 0.78 and an RMSE of 24.097 mg/L, and the ground validation gives an R^2^ of 0.840 and an RMSE of 30.062 mg/L. The SPM values predicted by the model and the measured values are distributed near the y = x line.

### 3.3. Consistency of SPM Concentration Retrieved from OLI and MSI Images

**Figure 4:**
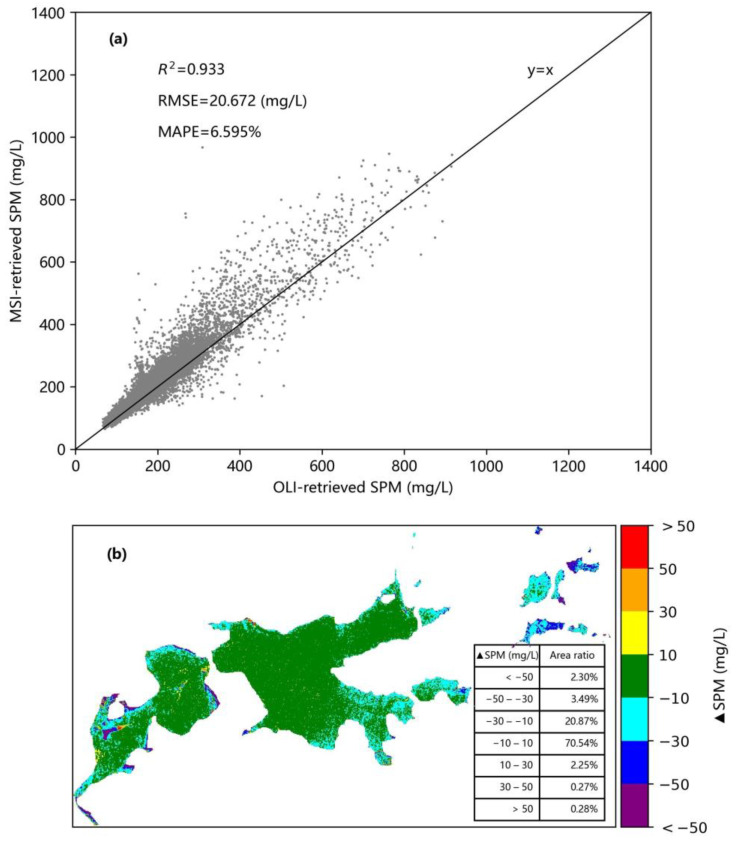
Comparison of SPM retrieval values from OLI and MSI images in Shengjin Lake using the same model: (**a**) Correlation of retrieval values, (**b**) difference of retrieval values.

Since MSI and OLI sensors have similar band settings and spectral response characteristics [25,29], the above-mentioned model was applied to the Sentinel-2 MSI and Landsat-8 OLI images acquired on 23 November 2019 in Shengjin Lake, and the consistency of the retrieved SPM concentration values was compared (Figure 4a,b).

It was found that the SPM values retrieved from OLI and MSI images that were acquired on the same day are highly consistent and distributed around the y = x line. The values of the accuracy measures are R^2^ = 0.933, RMSE = 20.672 mg/L, and MAPE = 6.595% (Figure 4a). The difference between the values of the SPM concentration retrieved from these two images is between −10 and 10 mg/L over 70.54% of the lake (Figure 4b). In the upstream and downstream parts of Shengjin Lake (which together account for 20.87% of the lake), the SPM concentration retrieved from the MSI image is higher than that retrieved from the OLI image, with the difference being between −30 and −10 mg/L. The difference between the two SPM concentration products is mainly related to the spatial distribution of SPM concentration in Shengjin Lake. Our previous studies have shown that the high SPM values of the lake are mainly concentrated in the whole downstream part and the south of the upstream part [36].

### 3.4. Universality of the Consistency of Two SPM Concentration Products

**Figure 5:**
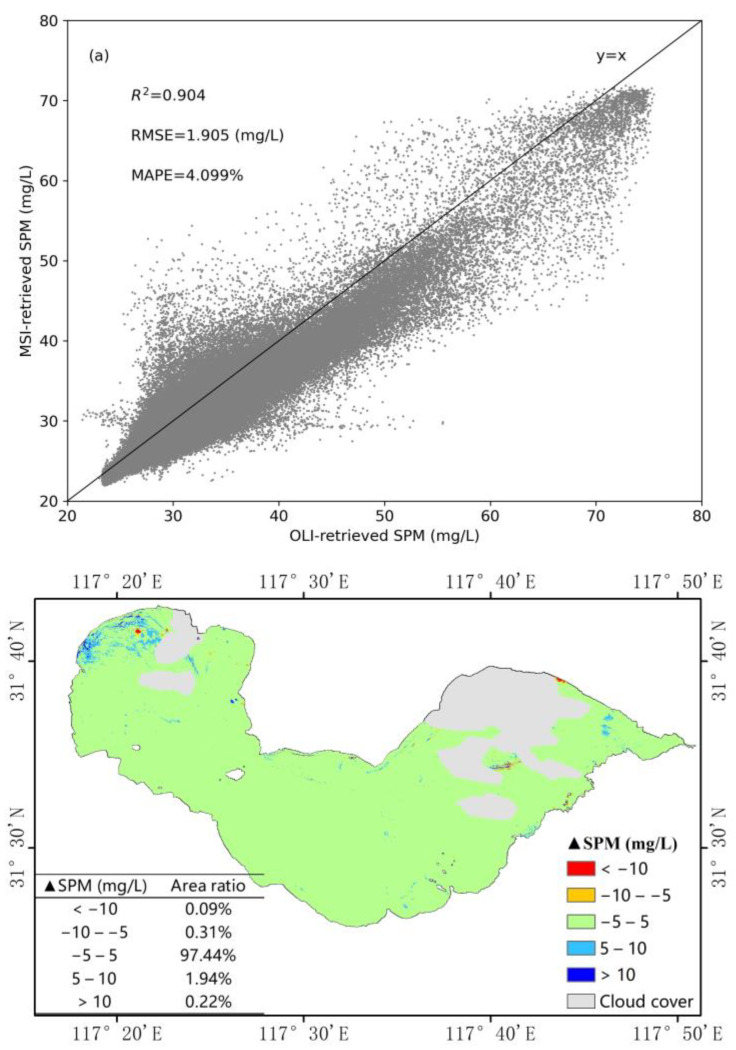
Comparison of SPM retrieval values from OLI and MSI images in Chaohu Lake, using the same model: (**a**) Correlation of retrieval values, (**b**) difference of retrieval values.

Based on spectral measurements made at Chaohu Lake on 27 December 2019, Rrs values for the MSI sensor were simulated using the method described in Section 2.4. The SPM retrieval model common for the OLI and MSI sensors was built based on the measured Rrs and SPM concentration values. The optimal SPM retrieval model can be expressed as y = 439.108 x^0.338^, where x is B8a × B4, R^2^ = 0.791, RMSE = 6.482 mg/L (Supplementary Figure S1). This model was used to retrieve the SPM concentration values from OLI and MSI images acquired on 23 November 2019, and the consistency of the retrieved SPM concentration values was compared in Chaohu Lake (Figure 5a,b).

It can be seen that the concentration values retrieved from the OLI and MSI images of Chaohu Lake are evenly distributed near the y = x line, and the accuracy can be expressed as R^2^ = 0.904, RMSE = 1.905 mg/L, MAPE = 4.099% (Figure 5a). The value of R^2^ for Chaohu lake is slightly lower than that for Shengjin Lake, but its MAPE value is slightly higher. Figure 5b shows that, over 97.44% of the lake, the difference between the values of the SPM concentration retrieved from these two images is between −5 and 5 mg/L. Over a small part of the lake (accounting for 1.94% of the area), the difference is between 5 and 10 mg/L. The above results indicate that the two SPM remote sensing products obtained from Landsat-8 OLI and Sentinel-2 MSI images by the same retrieval model also have good consistency in other turbid water regions.

## 4. Discussion

### 4.1. Influence of Atmospheric Correction on the Consistency of SPM Concentration Products

As a key element of the process of retrieving water quality parameters from satellite images, atmospheric correction (AC) has an important effect on the consistency of the Rrs values [20], as well as SPM concentration values retrieved from OLI and MSI images. First, the four common AC algorithms (MUMM, Atmospheric Correction for OLI’lite’(ACOLITE), Second Simulation of Satellite Signal in the Solar Spectrum (6S), Landsat 8 Surface Reflectance Code & Sen2cor (LaSRC & Sen2cor)) were applied to MSI and OLI images of Shengjin Lake acquired on 23 November 2019, and the derived Rrs values for each band were compared. From the comparison results obtained in our study (Table 2), it can be seen that the maximum MAPE of Rrs values using the same algorithm is 29.317%, while that using the different algorithm can be as high as 46.680%, which implies that the same AC algorithm is the premise for the consistency of the Rrs values from these two images. Among the four AC algorithms, the consistency of the Rrs values retrieved by the water AC algorithms (MUMM, ACOLITE) was significantly higher than that by the land AC algorithms (6S, LaSRC & Sen2cor), and the consistency of the Rrs values retrieved by MUMM algorithm (the MAPE is 1.910–10.587%) is generally higher than that by ACOLITE algorithm (the MAPE is 1.544–16.648%).

Then, the same SPM retrieval model was applied to the Rrs images derived from MSI and OLI images by the above four AC algorithms, and the retrieved SPM concentration values were compared (Table 2). The comparison results also show that among the four atmospheric correction algorithms, the SPM values obtained by the MUMM algorithm have the highest consistency and the smallest value difference (R^2^ = 0.933, RMSE = 20.672mg/L, MAPE = 6.595%), followed by the ACOLITE algorithm, while the LaSRC&Sen2Cor algorithm has the largest difference in SPM values. This phenomenon implies that the water atmospheric correction algorithm has a non-negligible influence on the consistency of SPM concentration products. Therefore, this paper used the MUMM algorithm to perform atmospheric correction on the Sentinel-2 MSI and Landsat-8 OLI images.

### 4.2. Influence of Retrieval Models on the Consistency of SPM Concentration Products

It can be seen that the same retrieval model has good consistency in SPM concentration from MSI and OLI images (Section 3.3 and Section 3.4). In Minnesota of the United States, the Yellow River estuary in China, and other regions, previous studies have also shown that the values of water quality parameters retrieved using the same model have good consistency [13,21]. Considering the regional differences in optical properties of inland waters, this study further explored the influence of the retrieval model on the consistency of SPM products in high-turbidity lakes.

#### 4.2.1. Optimal Models with Different Forms

Band combinations that had R > 0.8 (Table 1) and the corresponding SPM concentration values were used to develop the independent retrieval models for MSI and OLI sensors, based on the method described in Section 2.4. Comparing the calibration accuracy and validation accuracy for the different independent retrieval models, it was found that the optimal model for the MSI sensor was a quadratic polynomial model given by y = 537.223 x^2^ − 166.192x + 22.967, where x is B6/B3, R^2^ is 0.884, and RMSE is 25.862 mg/L (Supplementary Figure S2). The optimal model for the OLI sensor was found to be a power function model given by y = 613.417 x^1.709^, where x is B5/B3, R^2^ is 0.835, and RMSE = 30.584 mg/L (Supplementary Figure S3 and Table S1).

**Figure 6:**
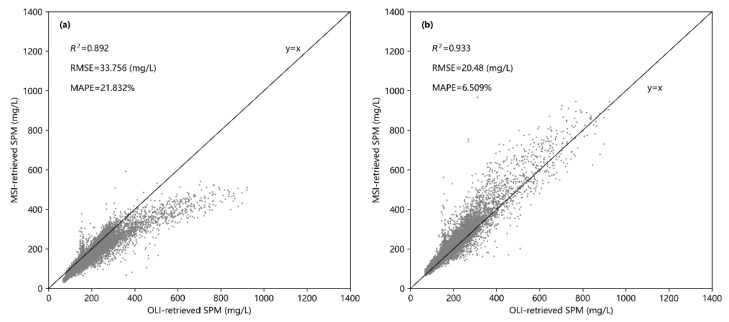
Correlation of SPM values retrieved from OLI and MSI images in Shengjin Lake using different models: (**a**) the optimal models with different forms, (**b**) the same form of models.

**Figure 7:**
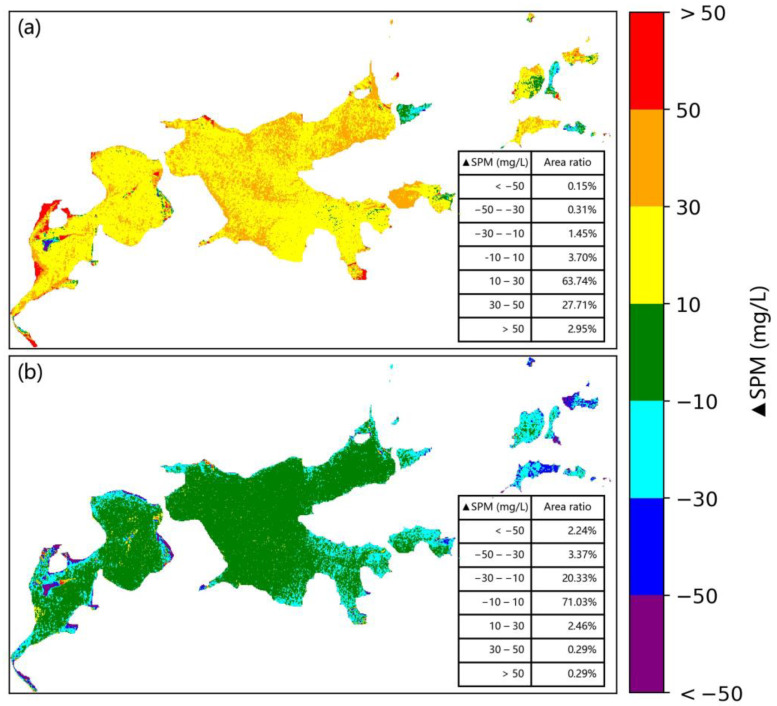
Difference between SPM values retrieved from OLI and MSI images in Shengjin Lake using different models: (**a**) the optimal models with different forms, (**b**) the same form of models.

These two SPM retrieval models with different forms were then applied to the MSI and OLI images of Shengjin Lake acquired on 23 November 2019. The consistency between the SPM concentration values retrieved from these two images were R^2^ = 0.892, RMSE = 33.576 mg/L, and MAPE = 21.832%. This implies that the consistency of the SPM concentration values retrieved from the MSI and OLI images using the two optimal models with different forms was obviously lower than that achieved by applying the same model to the two types of imagery (Figure 6a). In general, it was found that the SPM concentration values retrieved from the OLI image were higher by ~10–50 mg•L^−1^ than that retrieved from the MSI image. The regions with the SPM different values (ΔSPM) in the range of 10–30 and 30–50 mg•L^−1^ account for 63.74% and 27.71% of the area of Shengjin lake, respectively (Figure 7a).

#### 4.2.2. The Same form of Models

To develop the SPM retrieval models with the same form, the parameters of retrieval models were firstly recalibrated for MSI and OLI sensors based on the method described in Section 2.4, before applying to MSI and OLI images. The model for the MSI sensor was reconstructed as y = 610.210x^1.705^ (where x is B8a/B3, R^2^ = 0.840, RMSE = 30.62 mg/L) and that for the OLI sensor as y = 613.417x^1.709^ (where x is B5/B3, R^2^ = 0.835, RMSE = 30.584 mg/L). Then, these two models were applied to the OLI and MSI images of Shengjin Lake acquired on 23 November 2019. The correlation between the SPM concentration values retrieved from the two types of imagery was analyzed and the difference (OLI minus MSI) was also calculated. Using these retrieval models, it was found that the consistency between the SPM concentration value derived from these two images can be given by R^2^ = 0.933, RMSE = 20.48 mg/L, and MAPE = 6.509% (Figure 6b), which is slightly higher than that for the SPM concentration values retrieved from OLI and MSI images using the same model (see Section 3.3). The spatial distribution of the difference in the SPM concentration values derived from the two types of imagery was mostly consistent with that found by applying the same model: the region with the difference values (ΔSPM) in the range of −10 to 10 mg/L accounted for 71.03% of the area of Shengjin lake (Figure 7b), which is a slightly higher than that by applying the same model to two types of images. The above results show that SPM concentration values in turbid water retrieved from the OLI and MSI images were highly consistent when using either the same retrieval model or the models with the same form.

Combining satellite remote sensing technology with mathematical modeling has proven the great potential for the estimation of water quality parameters (i.e., SPM, colored dissolved organic matter (CDOM), chlorophyll-a) on the large scale more efficiently [19,20,37]. The SPM retrieval models suitable for Sentinel-2 MSI and Landsat-8 OLI sensors in this paper were constructed, based on the simultaneous measurements of SPM concentration and remote sensing reflectance. Besides the SPM, other material constituents in the water (such as phytoplankton pigments, yellow substances) can also affect its spectral characteristics [37,38]. The method proposed in this paper can be applied to the direct estimation of optically active parameters (i.e., chlorophyll-a, transparency, turbidity), or indirect estimation of non-optical active parameters (such as total nitrogen and total phosphorus) by constructing their correlation with the optically active constituents. Thus, our conclusions can provide a theoretical basis for the seamless integration of these two remote sensing water quality products in order to generate long time-series of remote sensing products for inland water.

## 5. Conclusions

In this paper, two turbid lakes located in the Yangtze River basin of China were selected as the study area, and the SPM retrieval models for Sentinel-2 MSI and Landsat-8 OLI sensors were constructed based on the measurement of Rrs and SPM concentration. The consistency between the SPM concentration values derived from OLI and MSI images was analyzed, and the influences of the AC algorithms and retrieval models on the consistency of the two SPM products was also explored.

An SPM retrieval model common for OLI and MSI sensors (y = 610.210x^1.705^) was constructed, which consisted of a power function model with B8a/B3 as the independent variable. The SPM retrieval model was applied to the OLI and MSI images of Shengjin Lake that were acquired on the same day, and the SPM values retrieved from these two images were found to be highly consistent. Then, the same method was applied to verify the consistency of the two SPM products in Chaohu Lake, and the similar results were obtained.

Following AC using the MUMM algorithm in SeaDAS software, the consistency between the Rrs values derived from the OLI and MSI images was high (R^2^ > 0.8, MAPE < 5%), except for the coastal blue and red bands, which is a prerequisite for ensuring the consistency of the SPM concentration values. In terms of the consistency of SPM concentration values from the OLI and MSI images, the MUMM algorithm in SeaDAS software performed better than other AC algorithms (ACOLITE, 6S, Sen2cor).

For the OLI and MSI images, the consistency of the SPM concentration values retrieved using the same form of models (R^2^ = 0.933, RMSE = 20.48 mg/L, MAPE = 6.509%) and using the same model (R^2^ = 0.933, RMSE = 20.672 mg/L, MAPE = 6.595%) were obviously higher than that achieved by applying the optimal models with different forms (R^2^ = 0.892, RMSE = 33.576 mg/L, MAPE = 21.832%). The consistency of the SPM concentration retrieved using the retrieval models with the same form was slightly higher than that using the same retrieval model.

## Figures and Tables

**Table 1:** Band combinations with correlation coefficient (R) greater than 0.8.

Sensor	Ratios	R	Sensor	Ratios	R
MultiSpectral Imager (MSI)	B8/B1	0.831	Operational Land Imager (OLI)	B5/B1	0.829
	B6/B2	0.835			
	B7/B2	0.835			
	B8/B2	0.859		B5/B2	0.851
	B6/B3	0.853			
	B7/B3	0.858			
	B8/B3	0.872		B5/B3	0.870
	B7/B5	0.824			

**Table 2:** Comparison of Rrs and SPM values from OLI and MSI images under different atmospheric correction algorithms.

AC	MUMM			ACOLITE			6S			LaSRC & Sen2cor		
	R^2^	RMSE	MAPE	R^2^	RMSE	MAPE	R^2^	RMSE	MAPE	R^2^	RMSE	MAPE
RrsB1	0.852	0.0019	10.492%	0.88	0.0020	16.648%	0.887	0.0044	29.317%	0.131	0.0057	46.680%
RrsB2	0.92	0.0005	1.910%	0.935	0.0006	2.956%	0.932	0.0012	6.122%	0.601	0.0016	7.475%
RrsB3	0.976	0.0007	2.032%	0.981	0.0005	1.544%	0.978	0.0026	9.494%	0.914	0.0010	3.032%
RrsB4	0.968	0.0024	10.587%	0.974	0.0021	9.516%	0.971	0.0037	17.562%	0.939	0.0028	12.786%
RrsB5	0.935	0.0007	3.323%	0.949	0.0007	4.416%	0.950	0.0007	5.137%	0.880	0.0022	23.341%
SPM	0.933	20.672	6.595%	0.948	22.66	9.365%	0.95	22.838	11.531%	0891	41.534	28.624%

## Data Availability

The data presented in this study are available on request from the corresponding author.

## Supplementary Materials

The following are available online at https://www.mdpi.com/1424-8220/21/5/1662/s1, Figure S1: Accuracy assessment of SPM retrieval model for OLI and MSI sensors in Chaohu Lake. (a) calibration accuracy, (b) validation accuracy; Figure S2: Accuracy assessment of SPM optimal model for MSI sensor in Shengjin Lake. (a) calibration accuracy, (b) validation accuracy; Figure S3: Accuracy assessment of SPM optimal model for OLI sensor in Shengjin Lake. (a) calibration accuracy, (b) validation accuracy; Table S1: SPM retrieval models constructed for OLI sensor in Shengjin Lake.
