# Supplementary material for: Consistency of Suspended Particulate Matter Concentration in Turbid Water Retrieved from Sentinel-2 MSI and Landsat-8 OLI Sensors
Source: Sensors (Basel). 2021 Feb 28;21(5):1662. doi: 10.3390/s21051662 (PMC7957495; doi:10.3390/s21051662)
Supplement: Supplementary file 1 [file sensors-21-01662-s001.pdf]

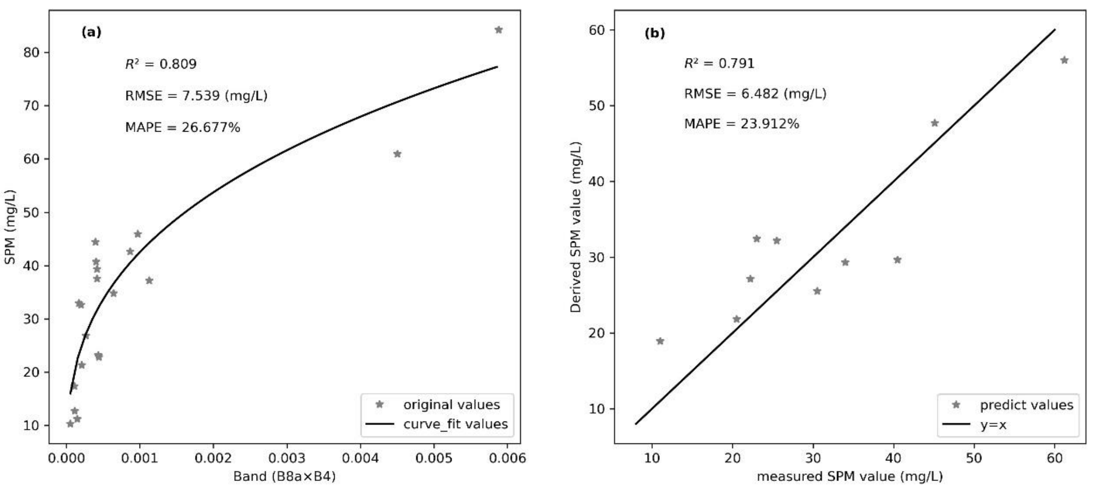

Figure S1: Accuracy assessment of SPM retrieval model for OLI and MSI sensors in Chaohu Lake. (a) calibration accuracy, (b) validation accuracy

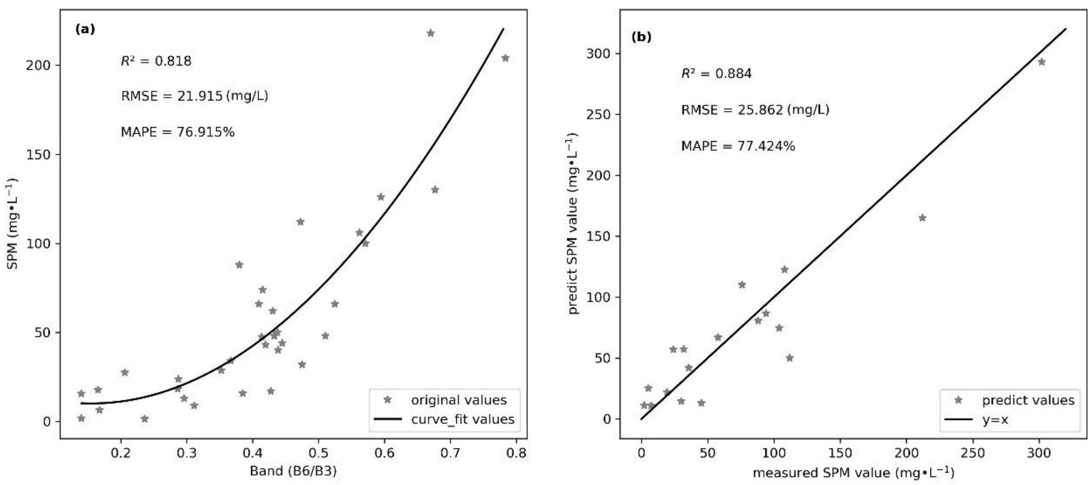

Figur S2. Accuracy assessment of SPM optimal model for MSI sensor in Shengjin Lake. (a) calibration accuracy, (b) validation accuracy

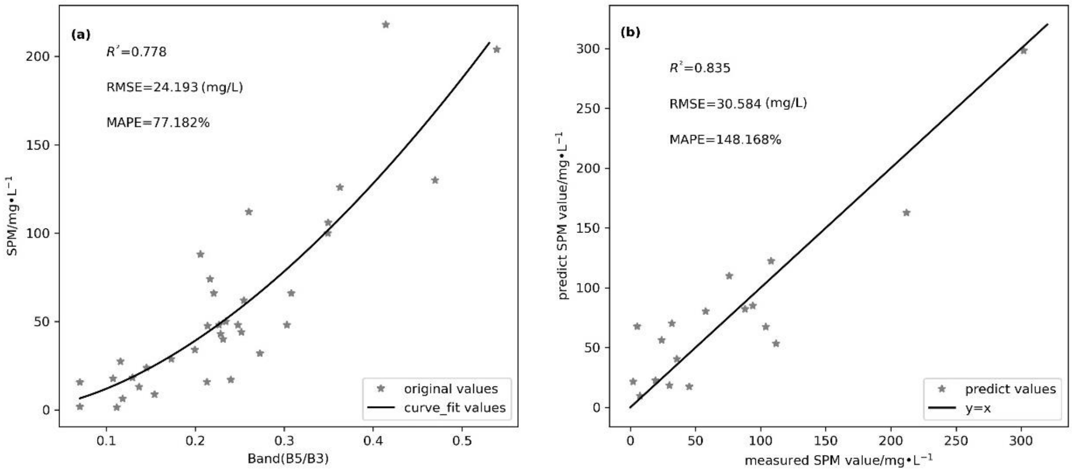

Figure S3. Accuracy assessment of SPM optimal model for OLI sensor in Shengjin Lake. (a) calibration accuracy, (b) validation accuracy

Table S1. SPM retrieval models constructed for OLI sensor in Shengjin Lake

**Band Ratios**	**Retrieval models**	**Calibration accuracy**			**Validation accuracy**		
		R^2	RMSE	MAPE	R^2	RMSE	MAPE
B5/B2	$y=292.497x-49.780$	0.713	30.37	91.93%	0.739	36.208	208.24%
B5/B2	$y=170.175x^2+132.479x-18.152$	0.725	29.727	86.81%	0.787	33.377	191.56%
B5/B2	$y=289.586x^{1.700}$	0.725	29.752	104.01%	0.791	33.234	193.95%
B5/B2	$y=570.621e^{-0.877/x}$	0.723	29.982	66.49%	0.717	37.505	183.27%
B5/B3	$y=451.792x-47.036$	0.778	26.703	81.24%	0.726	36.966	171.14%
B5/B3	$y=585.077x^2+120.599x-8.473$	0.805	25.043	66.26%	0.797	33.089	154.10%
B5/B3	$y=15.414e^{5.128x}$	0.765	27.725	138.34%	0.789	46.793	190.96%
**B5/B3**	$\mathbf{y=613.417x^{1.709}}$	**0.778**	**24.193**	**77.18%**	**0.835**	**30.584**	**148.17%**
B5/B3	$y=622.562e^{-0.590/x}$	0.805	25.331	49.41%	0.718	37.445	152.48%
